# Photothermal Transport for Guiding Nanoparticles Through the Vitreous Humor

**DOI:** 10.1002/advs.202516534

**Published:** 2025-12-22

**Authors:** Léa Guerassimoff, Yera Ussembayev, Louise De Clerck, Deep Punj, Martijn van den Broek, Filip Beunis, Katrien Remaut, Kevin Braeckmans, Stefaan C. De Smedt, Félix Sauvage

**Affiliations:** ^1^ Laboratory of General Biochemistry and Physical Pharmacy Faculty of Pharmaceutical Sciences Ghent University Ghent Belgium; ^2^ Liquid Crystals and Photonics Group Faculty of Engineering and Architecture Ghent University Ghent Belgium

**Keywords:** convection, drug delivery, indocyanine green, intravitreal injection, nanomedicines, photothermal transport, pulsed‐lasers, thermophoresis, vitreous

## Abstract

Visual impairments affect over 2.2 billion people worldwide, yet delivering drugs to the posterior segment of the eye, including the retina, remains a major challenge. Intravitreal injection, the standard administration route, often results in suboptimal drug diffusion through the vitreous, limiting drug access to the retina. While various strategies have been explored to enhance the mobility of drug molecules and nanomedicines (drugs encapsulated in nanoparticles) in the vitreous, no method has demonstrated *guided transport*. Here, we investigate photothermal transport of nanoparticles in the vitreous using a pulsed laser and indocyanine green, both clinically approved modalities. We show that photothermal transport guides nanoparticles from one location in the vitreous toward the laser‐illuminated area, away from the injection spot. Multiple‐particle tracking and numerical simulations reveal that this motion is predominantly driven by thermal convection, with thermophoresis contributing to a lesser extent. We identified parameters for optimization, including dye concentration, particle size, distance from the laser focus, and laser fluence. These findings establish a novel and clinically relevant paradigm for light‐guided drug delivery in the eye. To our knowledge, this is the first demonstration of guided light‐controlled particle transport in the vitreous using ocular dyes and pulsed lasers routinely applied in ophthalmology.

## Graphical abstract

Nanosecond laser irradiation of the clinically approved dye indocyanine green (ICG) enables light‐guided nanoparticle transport within the vitreous humor. The combined use of ICG and pulsed laser illumination induces localized convection and thermophoresis, directing particles toward illuminated regions. This study demonstrates controllable photothermal transport within the vitreous using clinical modalities, opening avenues for light‐guided drug delivery in ophthalmology.

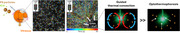

## 1 Introduction

Visual impairments affect more than 2.2 billion people worldwide, with refractive errors (RE), cataract (CAT), diabetic retinopathies (DR), glaucoma (GL), and age‐related macular degeneration (AMD) as the most frequent disorders [1, 2]. For many eye diseases, successful treatment remains a significant challenge. This is partially explained by the fact that drug delivery to specific compartments in the eye is still extremely difficult [3] due to the presence of various ocular barriers (e.g., lens, corneal barriers, vitreous humor, inner limiting membrane (ILM)) and poor control over the location where the drug ends up following administration. The main drug administration route to treat the posterior segment of the eye (including the retina) is intravitreal (IVT) injection, being the injection of drugs into the vitreous humor. As an example, anti‐VEGF antibodies are routinely intravitreally‐injected for the treatment of AMD [3, 4]. However, there is still an unmet need for delivery strategies that allow ‘modern’ medication, including antibodies and nanomedicines, to precisely reach the retina. Critical challenges persist following IVT injections, particularly in ensuring adequate drug diffusion through the vitreous humor and effective accumulation in the retina.

The vitreous humor is a gel‐like structure [5, 6], filling the space between the lens and the retina, which is considered a key structural component of the eye as it helps maintain its shape and protects it from mechanical damage [7]. When the eye has reached its adult size (typically between 14 and 18 years), this transparent and highly‐hydrated extracellular matrix is composed of two phases: [8] (i) a bulk gel part representing 80 % of the total volume of the vitreous humor, and (ii) a liquid phase [9]. The vitreous mainly consists of water (98–99 vol. %) [7, 10] and a network of biomacromolecules composed of collagen fibers, hyaluronan, and proteoglycans [5]. This 3D collagen network, which has been characterized by a mesh diameter of around 500 nm [9, 11], significantly contributes to the viscosity of the vitreous humor. Indeed, collagen fibers can vary in density, length, and texture, as can the ratio of collagen to hyaluronan, both of which significantly affect the viscosity of the vitreous [9].

As a dynamic barrier, the vitreous humor undergoes partial liquefaction with aging, changing its physicochemical and mechanical properties [12]. Four different states have been identified [12]: (i) an initial phase in which the vitreous remains a homogeneous gel‐like substance; (ii) the onset of liquefaction characterized by progressive dehydration of the gel and formation of isolated liquid pockets; (iii) extensive liquefaction during which these pockets enlarge and coalesce; and (iv) post vitreous detachment (PVD) which occurs as a result of vitreous liquefaction combined with the weakening of the vitreoretinal adhesion. While barely studied as of today, structural changes in the aging vitreous may significantly influence drug distribution following IVT injection, as the drug molecules may tend to follow connective flow patterns within the liquefied regions [9]. Moreover, the dense macromolecular matrix of the vitreous hinders diffusion, particularly for larger molecules (such as biotherapeutics) and nanomedicines [11, 13]. Additionally, drug reflux [1] and ‘multi‐directional diffusion’ from the injection site within the vitreous may further compromise drug delivery, substantially decreasing the probability of the drug reaching the retina.

Developing innovative strategies to enhance drug accumulation and penetration into the retina following IVT injection remains a significant challenge, while such strategies could significantly help in retinal disease management. Improvements in retinal drug delivery can be achieved through two main strategies. One strategy is the design of (drug‐loaded) nanocarriers with surface properties that avoid their aggregation in the vitreous (which prevents them from reaching the retina); examples include micelles [14], liposomes [15], and lipid nanoparticles [16]. Another strategy is based on the application of external stimuli into the vitreous to actively ‘guide’ drugs from the injection spot toward the retina. Delivering IVT‐injected therapeutics to a specific region of the retina based on external stimuli has, however, rarely been reported. One study reports on light‐driven nitrogen‐doped TiO_2_ nanomotors [17] moving in the vitreous while another study describes helical magnetic micro‐propellers whose motion in the vitreous can be controlled by a rotating magnetic field [18]. While these are praiseworthy efforts, the design and synthesis of micro‐propellers and nanomotors remain rather complex, while their ocular safety remains unclear. Therefore, exploring strategies that could safely enable the motion of molecules (like biotherapeutics) and nanoparticles (like nanomedicines) in the vitreous, using materials and external stimuli approved for clinical use in the vitreous, remains highly attractive.

**FIGURE 1:**
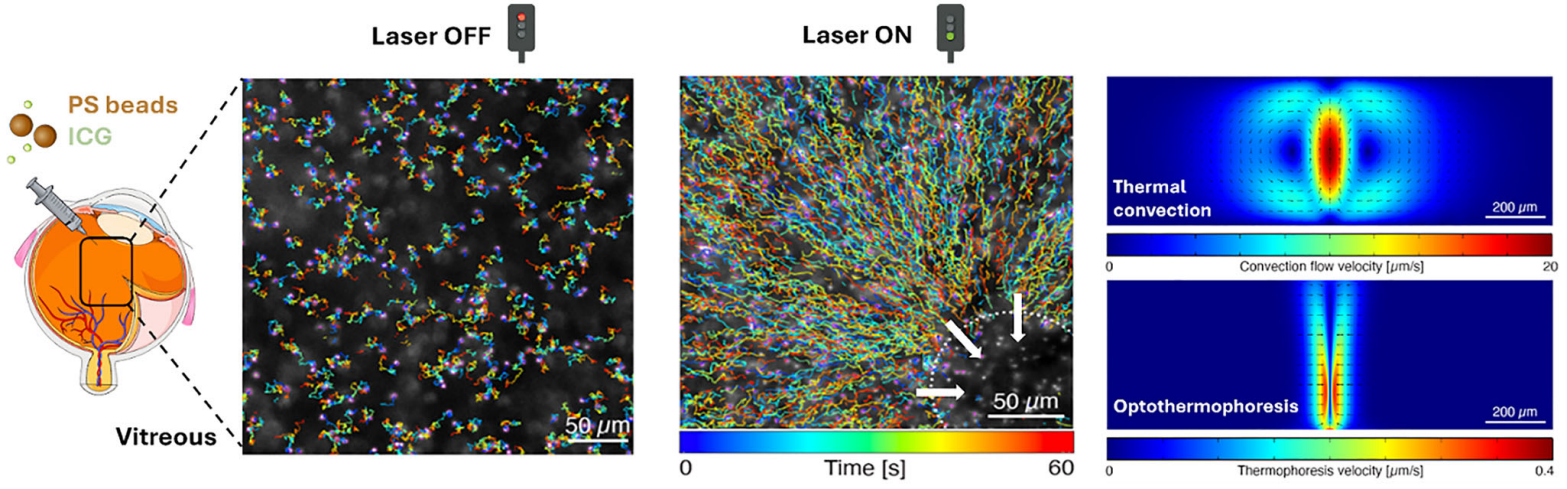
Schematic representation of photothermal transports, optothermophoresis, and thermal convection, to guide polystyrene (PS) nanoparticles through the vitreous. PS nanoparticles and the ocular dye indocyanine green (ICG) are added to the medium (water or vitreous), which is locally irradiated with a nanosecond pulsed‐laser. The left panel shows the random Brownian motion of nanoparticles when the laser is off. The middle panel shows the directed motion of nanoparticles when the laser is on: nanoparticles moved toward the laser‐illuminated spot (indicated by the white circle). The right panel shows numerical simulations of the two mechanisms contributing to photothermal transport in the vitreous: thermal convection (top), which is the dominant mechanism and reaches velocities up to ∼20 µm/s, and optothermophoresis (bottom), which provides only a minor contribution, with velocities up to ∼0.4 µm/s.

Photothermal transport is the phenomenon in which molecules or particles in a medium (fluid) move due to a local temperature increase of the medium induced by light absorption. This motion results from a combination of mechanisms, the primary ones being (i) thermal convection, (ii) Marangoni effect (i.e., mass transfer along an interface between two phases due to a gradient of the surface tension), and (iii) thermophoresis [19, 20, 21]. Thermal convection [22] occurs when heat causes density variations in a fluid. When the temperature of the fluid near a laser‐induced hot spot increases, the density lowers locally. This warmer, less dense fluid becomes buoyant and rises upward while the surrounding cooler (and denser) fluid moves downward. This results in a circulating continuous fluid motion, known as thermal convection, around the heated region. Unlike photothermal convection, opto‐thermophoresis [23] does not involve a bulk fluid motion, but a *directed* force acting on individual molecules/particles: the temperature gradient *around* each molecule/particle creates an imbalance in local intermolecular interactions, which drives the molecules/particles either toward or away from the heat source. This force has been explored to manipulate various objects like cells [24], particles [25], and DNA [26] in media like water [27] and cyclohexane [28]. Photothermal convection and opto‐thermophoresis have been mostly studied to enable the motion of molecules/particles on the surface of a light‐absorbing (solid) substrate (e.g., a plasmonic or metal surface) [27, 29], which is, however, challenging to realize in vivo. In the current study, we aim to explore the potential of light‐absorbing dyes as heating substrates for the induction of photothermal transport of nanoparticles (NPs) suspended in the solution. Next, we aim to elucidate if photothermal transport of nanoparticles can be achieved in the vitreous, using dyes and lasers that are currently in ophthalmological use. More specifically, we explore the potential of nanosecond pulsed‐lasers and vital dyes, such as indocyanine green (ICG) and trypan blue (TB), that are commonly used in the clinic for the staining of ocular tissues [30, 31] and for retinal angiography [32, 33]. We aim to provide a sound physical understanding of particle motion driven by photothermal transport in the vitreous, investigate this phenomenon experimentally using multiple‐particle tracking (MPT), and aim to reveal whether directed (‘guided’) transport of nanoparticles in the vitreous based on opto‐thermophoresis and/or thermal convection can be achieved (Figure 1). To the best of our knowledge, photothermal transport in biological matrices has never been reported. Also, while vital dyes and pulsed‐lasers are widely used in ocular interventions, their combined use in the eye is uncommon. The current study is part of our efforts [34] to widely explore the potential of a combined use of ocular dyes and pulsed‐lasers for novel treatments of eye diseases and advanced ocular surgeries. By demonstrating that localized and controllable convection and optothermophoresis can be achieved with modalities already used in the clinic, this work opens avenues for guiding intravitreal transport in a clinically translatable manner.

## 2 Results

### 2.1 Photothermal Transport of Polystyrene (PS) Nanoparticles in Water

**FIGURE 2:**
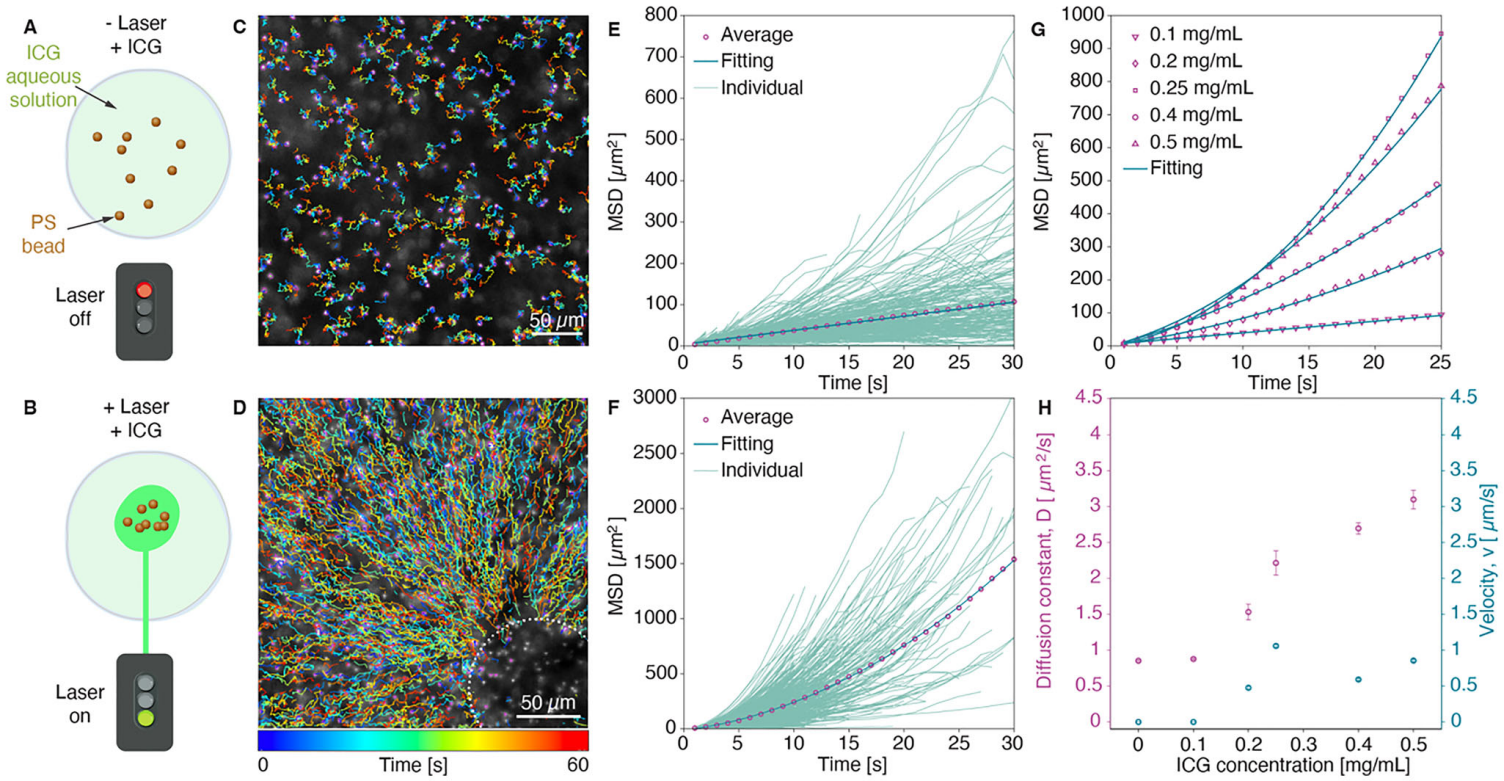
Photothermal transport of PS nanoparticles (d_p_ = 520 nm) in the presence of ICG upon laser irradiation in water. (A) Schematic illustration of PS nanoparticles mixed with ICG in water without laser exposure. (B) Schematic representation of PS nanoparticle transport under laser irradiation. (C) Representation of colored single traces of PS nanoparticles (dilution ratio 1/1000 (v/v)) from MPT analysis in water samples in the presence of ICG at 0.5 mg/mL and without laser irradiation (Video S3). (D) Representation of colored single traces of PS nanoparticles (dilution ratio 1/1000 v/v) from MPT analysis in water samples in the presence of ICG at 0.5 mg/mL and upon laser irradiation (2.07 J/cm^2^, 532 nm). The laser spot is located within the white circle. (Video S1). (E) Evolution of MSD values as a function of time obtained from MPT analysis of Video S3 (i.e., in water, PS nanoparticles at a dilution ratio of 1/1000 (v/v), ICG at 0.5 mg/mL, no laser irradiation). Representation of individual nanoparticle traces and implementation of a linear fitting model (diffusion regime). Number of traces for analysis = 609. (F) Evolution of MSD values as a function of time obtained from MPT analysis of Video S1 (i.e., in water, PS nanoparticles at a dilution ratio of 1/1000 (v/v), ICG at 0.5 mg/mL, upon laser irradiation (2.07 J/cm^2^, 532 nm)). Representation of individual particle traces and implementation of a quadratic fitting model. Number of traces for analysis = 2326. (G) Time traces of average MSD acquired for different ICG concentrations (i.e., 0, 0.1, 0.2, 0.25, 0.4, and 0.5 mg/mL) in water (PS nanoparticles at a dilution ratio of 1/5000 (v/v)) from the extracted individual time traces are summarized in Figure S2. (H) Evolution of extracted fitting parameters, the diffusion constant *D* (left *y*‐axis) and the particle velocity *v* (right *y*‐axis), as a function of ICG concentration (i.e., 0, 0.1, 0.2, 0.25, 0.4, and 0.5 mg/mL) in water (PS nanoparticles at a dilution ratio of 1/5000 (v/v)).

Laser pulse irradiation (532 nm, pulse duration 2–5 ns, 2.07 J.cm^−2^) in the presence of indocyanine green (ICG, 0.5 mg/mL) (Figure 2A) revealed the attraction of the 520 nm PS nanoparticles toward the laser beam focus (Figure 2B; Video S1, see Table S1) and an increase of their concentration within the illuminated zone (see Figure S1A). Interestingly, no attraction of PS nanoparticles toward/at the laser focus was observed in the absence of ICG (Video S2) or without laser irradiation (Video S3). This shows that photothermal particle motion depends on both the presence of the dye and laser exposure. Note that, under both control conditions (i.e., without ICG or without laser irradiation), PS nanoparticles still exhibited Brownian motion (Figure 2C).

To characterize the nature of particle motion, we recorded time‐lapse videos and applied multi‐particle tracking (MPT) analysis [11, 35, 36, 37] (Videos S1–S3). According to Einstein's relation, the diffusion coefficient *D* is given by:

(1) $$D=k_{B}\;T/\gamma$$

where *k_B_* = 1.38×10^−23^ J/K is the Boltzmann constant, *T* is the absolute temperature, and *γ* is the friction coefficient experienced by the particle in the fluid medium. For spherical particles, the friction coefficient *γ*, also known as the hydrodynamic drag, is related to the particle diameter *d_p_* and the temperature‐dependent dynamic viscosity *η* of the liquid by Stokes' law:

(2) $$\gamma =3\pi \eta d_{p}$$

To quantify particle trajectories, we tracked (see Methods) the 2D positions (*x*, *y*) of individual particles across time *t* from video frames (Figure 2C,D). From this tracking data, the mean‐square displacement (MSD) as a function of lag time *τ* was calculated as:

(3) $$\langle r^{2}\left(\tau \right)\rangle =\langle {\left(x\left(t+\tau \right)-x\left(t\right)\right)}^{2}+{\left(y\left(t+\tau \right)-y\left(t\right)\right)}^{2}\rangle$$

The MSD provides insights into the nature of particle motion—whether it is purely diffusive or influenced by an external force. In control experiments (i.e., Video S2: no ICG, no laser; Video S3: ICG, no laser), individual particle tracks display random, non‐directional Brownian motion (Figure 2C). This is confirmed by the MSD profile (Figure 2E) from Video S3, which fits well to a linear model for normal 2D diffusion [35]:

(4) $$\langle r^{2}\left(\tau \right)\rangle =4D\tau$$

From this fit, the experimental diffusion constant was calculated as *D*_exp_ = 0.853 ± 0.007 µm^2^/s, closely matching the theoretical value *D*_th_ = 0.825 µm^2^/s for particles of 520 nm diffusing in water at room temperature. In contrast, under laser irradiation and in the presence of ICG (Video S1), particle trajectories (Figure 2D) exhibit directed motion toward the laser focal point. The corresponding MSD (Figure 2F) deviates from the linear model and instead follows a quadratic dependence, indicative of active transport [35]:

(5) $$\langle r^{2}\left(\tau \right)\rangle =4D\tau +{\left(v\tau \right)}^{2}$$

Here, *v* represents the velocity corresponding to the directed motion of the particles. Fitting this model to MSD data yields a significantly higher apparent diffusion constant *D*_exp_ = 2.68 ± 0.12 µm^2^/s and a velocity *v* = 1.160 ± 0.021 µm/s, demonstrating the influence of the photo‐induced driving force on the motion of nanoparticles. Notably, the diffusion constant under laser irradiation is approximately 3 times higher than in control experiments (Video S3: ICG, no laser), due to the temperature rise and corresponding reduction in viscosity caused by the ICG absorption.

To gain deeper insight into the role of ICG in mediating photothermal transport of nanoparticles, we investigated a range of ICG concentrations: 0.1, 0.2, 0.25, 0.4, and 0.5 mg/mL, while keeping a constant PS particle concentration. The extracted individual time traces are shown in Figure S2, whereas the averaged MSD values are summarized in Figure 2G. Under pulsed laser irradiation (2.07 J/cm^2^, 532 nm), PS particles exhibit enhanced motion toward the laser focal point at high ICG concentrations (i.e., 0.25–0.5 mg/mL), in contrast to the slower or negligible motion at lower concentrations (i.e., 0.1–0.2 mg/mL; see Video S4 for 0.1 mg/mL). This trend is quantitatively illustrated in Figure 2H, where the extracted parameters from MSD analysis, including the diffusion constant *D* and particle velocity *v*, are shown as a function of ICG concentration. At concentrations below 0.2 mg/mL, the particle motion remains purely Brownian motion. However, starting from 0.2 mg/mL, we observe additional directed motion. Above this threshold, both diffusion and particle velocity increase, with particle velocities reaching up to about 1 µm/s. Based on the measured diffusion constants and Equations (1) and (2), we estimated the corresponding localized temperature (see Methods) increases for ICG concentrations of 0.1 and 0.5 mg/mL, which yield approximate temperatures of 20°C and 85°C, respectively. These results highlight the critical role of ICG in enabling laser‐induced photothermal transport of PS nanoparticles through water, along with the ability to tune particle velocity by simply varying the dye concentration.

### 2.2 Photothermal Transport of PS Particles in Bovine Vitreous

We next investigated and characterized the motion of PS nanoparticles (i.e., MSD regime, diffusion constant, and velocity) upon laser irradiation in the vitreous. Laser irradiation (532 nm, pulses of 2–5 ns) at different fluences (ranging from 0.34 to 1.03 J/cm^2^) of vitreous samples in the presence of ICG (0.5 mg/mL) first confirmed the attraction of the 520 nm PS nanoparticles toward the laser beam and their subsequent increased concentration in the illuminated zone (see Figure S1B; Video S5 for 0.69 J/cm^2^). Indeed, quantitative analysis (see Figure S1 B,C) shows that the mean gray intensity, corresponding to particle scattering, measured after laser irradiation is significantly higher than the one obtained before laser irradiation (〜1.4 vs. 1.05, respectively), confirming the efficient attraction of particles at the laser spot after irradiation. Control samples (i.e., without laser irradiation (Video S6, 0.69 J/cm^2^) or without ICG (Video S7, 0.69 J/cm^2^)) confirmed that the motion of particles was dependent on both the dye and light, as no particle attraction was observed in samples lacking ICG or laser irradiation. As temperature control is also critical for safety, we experimentally monitored the bulk temperature in vitreous samples under our experimental conditions (ICG 0.5 mg/mL; 532 nm, 0.69 J/cm^2^, 2–5 ns pulses). The temperature remained stable (around 19°C) during 120 s of laser irradiation (see Figure S3), indicating minimal laser‐induced heating in the bulk medium.

**FIGURE 3:**
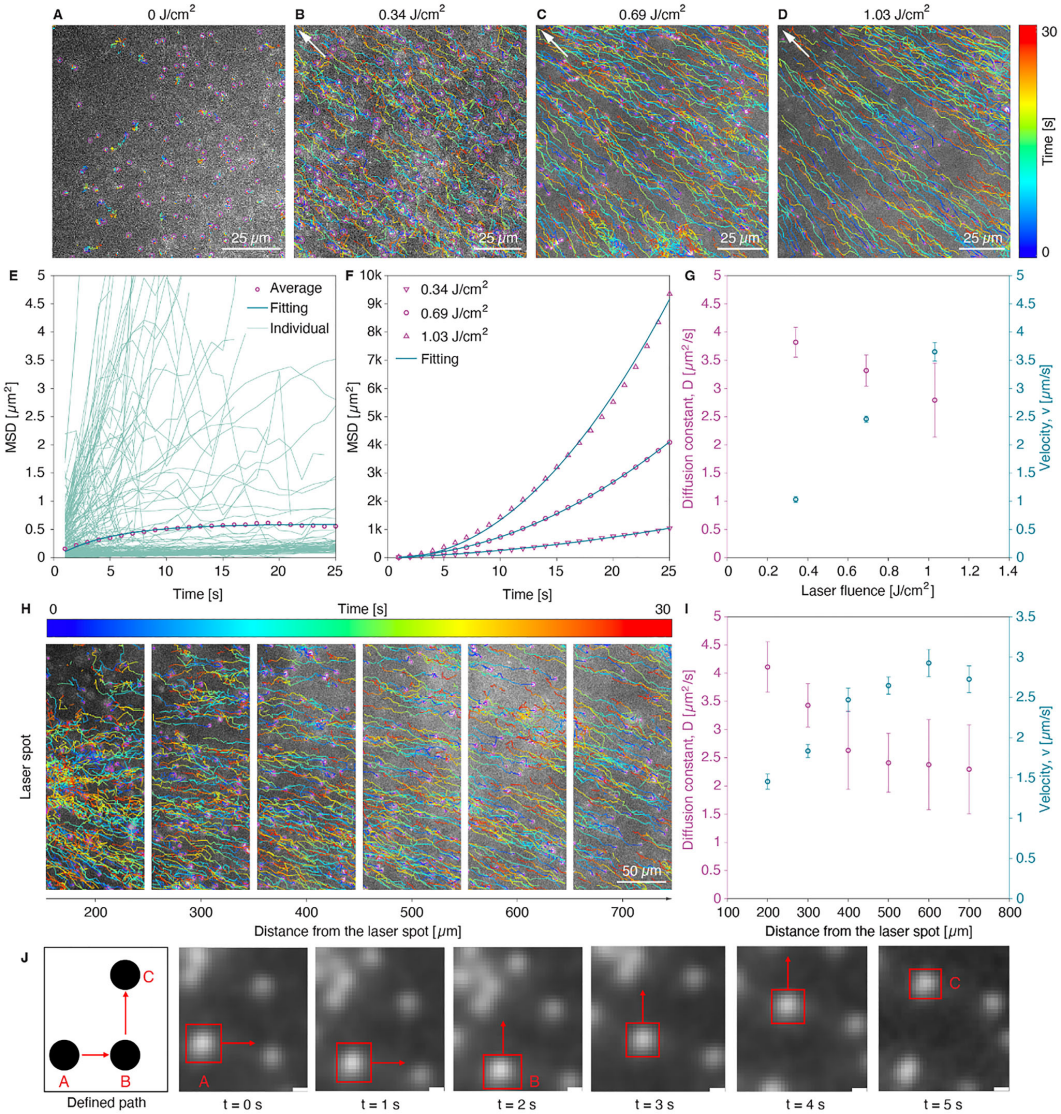
Photothermal transport of PS nanoparticles (d_p_ = 520 nm) with ICG upon laser irradiation in bovine vitreous. (A) Representation of colored single traces of PS nanoparticles (dilution ratio 1/1000 (v/v)) from MPT analysis of Video S6 in vitreous samples in the presence of ICG at 0.5 mg/mL and without laser irradiation. (B–D) Representation of colored single traces of PS nanoparticles (dilution ratio 1/1000 (v/v)) in vitreous samples in the presence of ICG at 0.5 mg/mL and with laser irradiation (laser spots are outside of the selected area of interest indicated with white arrows in the top left corner). MPT analysis has been performed on videos recorded at different laser fluences: 0.34, 0.69 (Video S5), and 1.03 J/cm^2^. (E) Evolution of MSD values with time from MPT analysis of Video S6. Representation of individual particle traces and exponential fitting model (i.e., confined motion). Number of traces for analysis = 447. (F) Time traces of average MSD acquired for different laser fluences extracted from the individual traces are summarized in Figure S4. (G) Evolution of the obtained fitting parameters, including diffusion constant *D* (left *y*‐axis) and particle velocity *v* (right *y*‐axis) as a function of laser fluence. (H) Representative colored trajectories of PS nanoparticles (dilution ratio 1:1000 (v/v)) located at distances ranging from 100 to 800 µm from the laser spot from MPT analysis of Video S8 in vitreous samples in the presence of ICG at 0.5 mg/mL. (I) Fitting parameters, diffusion coefficient *D* (left *y*‐axis) and particle velocity *v* (right *y*‐axis), plotted as a function of distance from the laser spot extracted from the MSD traces exemplified in Figure S5. (J) Representative frames extracted from Video S9 showing the controlled displacement of a polystyrene particle (520 nm) in bovine vitreous containing ICG (0.5 mg/mL). A nanosecond laser (532 nm, 0.69 J/cm^2^) irradiates the region within its beam, which is sequentially repositioned from location A to B and then to C. The tracked particle (highlighted by red boxes) follows the imposed “inverted L” trajectory of the moving laser spot (highlighted by red arrows), demonstrating directed and spatially selective manipulation. Scale bar: 3 µm.

Figure 3 shows multi‐particle tracking analysis of PS nanoparticles in bovine vitreous, highlighting two distinct regimes of particle motion depending on laser irradiation conditions, as demonstrated in Videos S5 and S6.

#### 2.2.1 Confined Motion Without Laser Irradiation (0 J/cm^2^)

In the absence of laser exposure (Video S6), PS nanoparticles exhibit confined, low‐mobility behavior. Individual particle trajectories appear confined in space and randomly oriented (Figure 3A), consistent with restricted Brownian motion. The mean square displacement curve (Figure 3E) shows a saturating trend, indicative of particle confinement. This behavior is described using an exponential confinement equation [35]:

(6) $$\langle r^{2}\left(\tau \right)\rangle =r_{c}^{2}\;\left(1-exp\left(-4D\tau /r_{c}^{2}\right)\right)$$

where *r_c_* is a confinement radius, defined as the characteristic spatial limit within which the particle remains trapped due to physical or environmental constraints (e.g., the mesh structure of the vitreous). It represents the maximum spatial extent of free diffusion before encountering restrictions. Fitting the expression (6) to the experimental data from Video S6 yields *r_c_* = 0.77 ± 0.06 µm and a diffusion constant *D*_exp_ = 0.029 ± 0.006 µm^2^/s, significantly lower than the expected diffusion coefficient in water (0.825 µm^2^/s).

#### 2.2.2 Directed Motion with Laser Irradiation (0.34–1.03 J/cm^2^)

Upon laser illumination (Video S5), PS nanoparticles exhibit clear directed motion toward the laser focus, forming elongated trajectories (Figure 3B–D) that become increasingly elongated with increasing laser fluence. The average MSD plots in Figure 3F are extracted from the individual traces shown in Figure S4 and display a pronounced quadratic time dependence, indicative of active transport. Figure 3F shows MSD curves for three laser fluence levels (0.34, 0.69, and 1.03 J/cm^2^), with steeper curves at higher fluences, reflecting faster directed motion. Figure 3G quantifies this trend, plotting the diffusion constants and velocities as a function of laser fluence. While the diffusion coefficient remains nearly constant, the particle velocity increases monotonically with the fluence, confirming the fluence‐dependent nature of laser‐induced transport (Table 1). Therefore, we have successfully demonstrated the photothermal transport of 520 nm nanoparticles in the presence of ICG in the vitreous humor, as well as its strong dependency on laser fluence values.

**TABLE 1:** Influence of the laser fluence on the photothermal transport of PS nanoparticles (i.e., diffusion constant and particle velocity) in bovine vitreous humor.

Laser fluence (J/cm^2^)^a^	Diffusion constant *D* (µm^2^/s)^b^	Particle velocity *v* (µm/s)^c^
0	0.0294 ± 0.006	‐^d^
0.34	3.82 ± 0.266	1.03 ± 0.052
0.69	3.32 ± 0.277	2.46 ± 0.054
1.03	2.79 ± 0.656	3.65 ± 0.165

^a^ determined from the formula: *Laser fluence (J.cm^−2^) = (Energy at the sample E_sample_ (J)) / (π x (laser beam diameter (cm)/2)* [2]) where E_sample_ = Delivered energy (J) x conversion factor, with a laser beam diameter of 217.4 µm and a conversion factor of 2.56.

^b^ obtained from Equation (6) for 0 J/cm^2^ and Equation (5) for 0.34, 0.69, and 1.03 J/cm^2^.

^c^ calculated from Equation (5).

^d^ no significant velocity has been observed without laser irradiation.

Next, we examined the photothermal transport of 520 nm‐ PS nanoparticles in the vitreous in the presence of ICG as a function of their distance from the laser spot. Multi‐particle tracking analysis of Video S8 was therefore performed in 100 µm wide regions, located 200–800 µm from the laser focal point, as shown in Figure 3H. The corresponding MSD traces are shown in Figure S5, whereas the extracted fitting parameters for each region, including diffusion constants and particle velocities, are summarized in Figure 3I. The diffusion coefficient of the PS nanoparticles in the vitreous decreases significantly from 4.1 ± 0.45 µm^2^/s to 2.6 ± 0.69 µm^2^/s between 200 and 400 µm from the laser spot, after which it plateaus even beyond 800 µm. This trend suggests reduced collagen network density and/or loss of structural integrity closer to the laser spot, likely due to localized heating that causes partial degradation of the surrounding collagen matrix. MSD analysis supports this observation, showing steeper slopes for particles within 100–400 µm from the laser (Figure S5A–C), indicating enhanced mobility. It is interesting to note that particle velocities, however, decrease as they approach the laser spot, dropping from 2.72 ± 0.17 µm/s at 700 µm to 1.46 ± 0.09 µm/s at 200 µm (Figure 3I). Overall, the obtained results indicate a substantial local temperature rise due to ICG absorption in the vitreous, resulting in enhanced particle diffusion at the laser focal point and driving nanoparticle transport over long distances (up to nearly one millimeter) from the heating site.

Figure 3J further demonstrates that the laser‐induced transport can be spatially redirected in a controlled manner. When the laser spot is manually displaced along a predefined trajectory (A → B → C), the corresponding thermal hotspot is repositioned within the vitreous. As shown in Video S9, a 520 nm particle consistently migrates toward each new hotspot location and follows the imposed “inverted L” path. This experiment shows that simply moving the laser focus is enough to redirect particle motion, enabling targeted and spatially selective particle manipulation in the vitreous.

**FIGURE 4:**
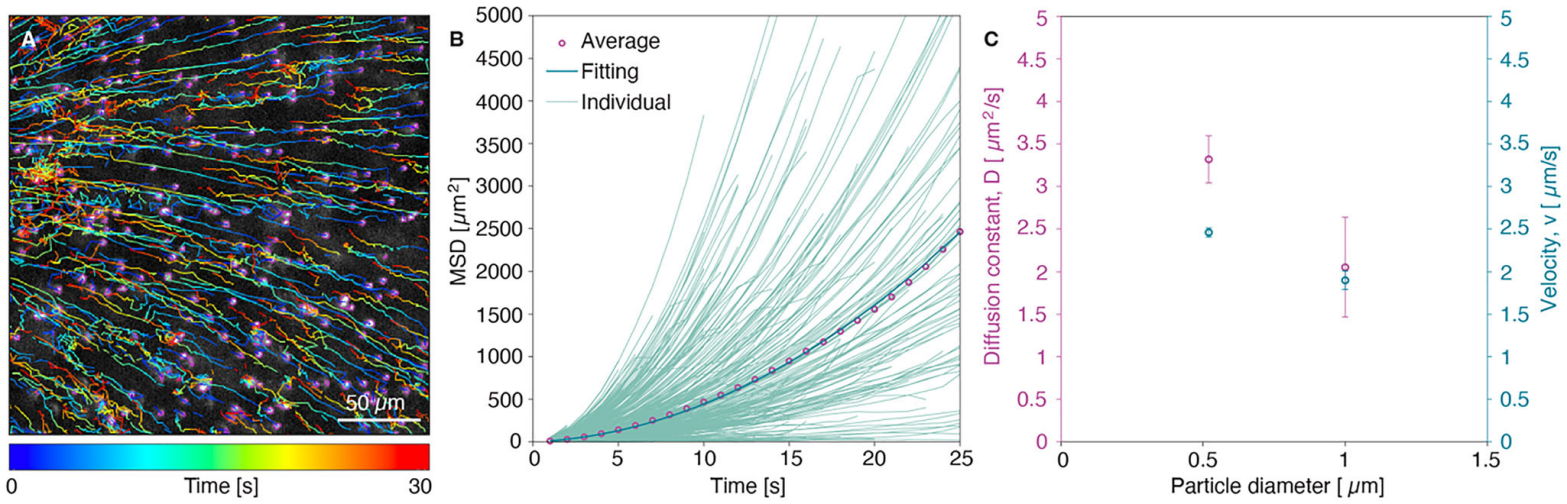
Photothermal transport of PS particles (d_p_ = 1 µm) in the presence of ICG upon laser irradiation in bovine vitreous. (A) Representation of colored single traces of PS particles (dilution ratio 1/1000 (v/v)) from MPT analysis of Video S10 in vitreous samples in the presence of ICG at 0.5 mg/mL upon laser irradiation (0.69 J/cm^2^, 532 nm). The laser spot is outside of the selected area of interest on the left‐hand side. (B) Evolution of MSD values with time from MPT analysis of Video S10 with individual particle traces and quadratic fitting model. Number of traces for analysis = 926. (C) The obtained fitting parameters, including diffusion constant *D* (left *y*‐axis) and particle velocity *v* (right *y*‐axis), as a function of the particle diameter.

To assess the impact of the diameter of the particles on their photothermal transport in the vitreous, we performed additional experiments using 1 µm PS particles. As the vitreous has a reported average mesh size of 510–565 nm [9, 11], particles exceeding this size become immobilized within the network [9, 11], raising the question of whether photothermal effects would suffice to induce their motion. Similar to smaller particles (520 nm), in the presence of ICG (0.5 mg/mL) and upon laser irradiation (0.69 J/cm^2^, 532 nm), these larger particles were also attracted toward the laser focal point, and their concentration increased at the illuminated zone (Figure 4A). MSD calculations obtained from MPT analysis of Video S10 confirmed a quadratic motion of 1 µm PS particles, relatively similar to the one observed with 520 nm PS particles, as shown in Figure 4B. However, the trajectory of these particles appears slightly irregular—compared to 520 nm particles—since they follow different paths throughout the vitreous, as shown by colored single particle traces in Figure 4A in comparison with elongated and straight trajectories obtained under the same conditions (Figure 3C). Upon laser irradiation (0.69 J/cm^2^, 532 nm), the motion of these larger particles was characterized by a lower diffusion constant and a comparable velocity to those measured for 520 nm particles (i.e., *D* = 2.05 ± 0.58 vs. 3.32 ± 0.277 µm^2^/s and *v* = 1.9 ± 0.11 vs. 2.46 ± 0.054 µm/s, respectively, Figure 4C), confirming the difficulty for larger particles to go through the network due to increased hydrodynamic drag.

### 2.3 Numerical Simulations of the Photothermal Effects

**FIGURE 5:**
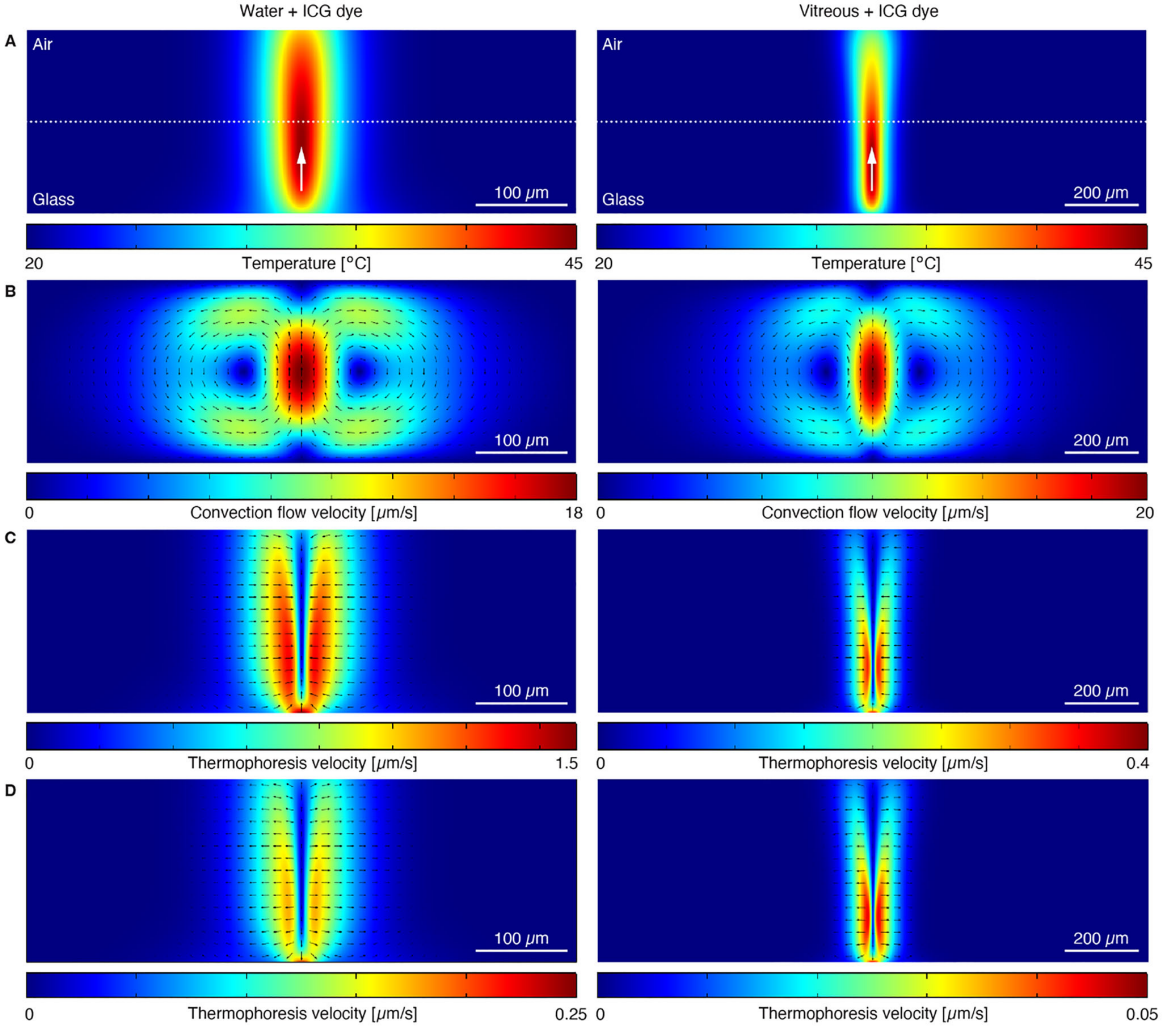
Numerical calculations of different photothermal effects induced by ICG absorption of the laser energy in water (left panels) and in bovine vitreous (right panels, x,z plane). (A) Steady‐state temperature distribution following laser irradiation shows localized heating centered around the laser beam, propagating upward as indicated by the white arrow and focused at the center of the sample, as marked by the dotted line. The samples are modeled between two boundaries, with air at the top surface and glass at the bottom; as a result, the temperature profiles are asymmetric along the *z*‐axis due to differing thermal conductivities of air and glass. (B) Thermal convection flow fields generated by the temperature gradient, with arrows indicating the direction and circulation pattern of fluid motion. (C) Thermophoretic velocities of particles due to opto‐thermal gradients (thermophoresis), with arrows indicating the particle motion toward the laser spot (thermophilic) for a negative Soret coefficient S_T_ < 0. (D) Thermophoretic velocities of particles for S_T_ > 0, where particles move away from the laser spot (thermophobic) due to repulsive thermophoresis.

To investigate the underlying mechanisms driving nanoparticle motion in water and in the vitreous, we simulated different photothermal effects using finite‐element modeling [38, 39, 40, 41] (see Methods). These simulations account for heat generation resulting from ICG light absorption under laser irradiation, and compute the resulting temperature gradients, fluid dynamics (thermal convection), and thermophoretic particle transport (Figure 5). The calculated results reveal strong contrasts between water and vitreous environments. In water, the laser‐induced heating produces a broad and intense temperature gradient (Figure 5A, left), leading to pronounced thermally‐induced convection flows (Figure 5B, left) with recirculating patterns and peak flow velocities up to ∼18 µm/s in the hot spot. These flows, coupled with thermophoretic forces which can speed up the particles up to 1.5 µm/s (Soret coefficient (S_T_) < 0 , Figure 5C, left) or slow down to 0.2 µm/s (S_T_ > 0 ; Figure 5D, left), effectively drive nanoparticles toward the laser focus, in good agreement with the experimentally observed directed motion. A similar effect is observed in *the vitreous* with localized temperature increase (Figure 5A, right) and more significant convective motion (Figure 5B, right) due to the thicker sample height. Several studies have reported that thicker samples have stronger convection flows under thermal gradients, which become dominant over thermophoretic motion [19, 23, 42, 43]. The calculated thermophoretic velocities in the vitreous are reduced (Figure 5C, right), reaching only ∼0.35 µm/s compared to 1.5 µm/s in water, caused by the higher liquid viscosity. Another notable feature is that the radius of particle attraction driven by thermal convection toward the heating spot scales with the sample thickness, with flow velocities attenuating over distances of approximately 200 µm in water and 500 µm in the vitreous (Figure S6), whereas thermophoretic forces act over much shorter ranges, typically less than 100 µm around the laser beam (Figure S6). Although the sign of the Soret coefficient (S_T_) may vary depending on experimental conditions, simulations performed with both S_T_ < 0 (see Figure 5C) and S_T_ > 0 (see Figure 5D) consistently show particle attraction within the laser beam region. This behavior primarily arises from the predominance of convective flows. Together, these findings indicate that particle transport in the vitreous is predominantly governed by convective fluid flow, with only a minor thermophoretic contribution.

Beyond the effects of temperature and viscosity, fluid velocity is also strongly influenced by spatial position relative to the heat source and nearby boundaries. As previously shown in Figure 5B, velocity fields vary with both the radial distance from the laser center and the vertical distance from the glass substrate. Figure S6 further illustrates this behavior, where extracted velocity profiles for water and vitreous reveal that particles closer to the glass surface (*z* = 10 µm) experience reduced convection velocities due to stronger viscous drag at the no‐slip boundary, whereas particles further away from the surface (*z* > 20 µm) move faster. This spatial dependence explains the variations in particle velocities observed experimentally in water (Figure 2H), where speeds fluctuate around 1 µm/s for ICG concentrations between 0.25 and 0.5 mg/mL. These differences likely result from variations in the focal plane position relative to the glass surface during microscopy, as well as the distance between the analyzed region and the laser focal spot (approximately 100–200 µm), as depicted in Figure S6.

### 2.4 Photothermal Transport of PS Nanoparticles in Aging Vitreous

**FIGURE 6:**
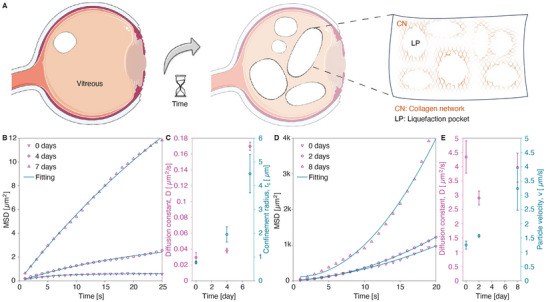
Influence of vitreous liquefaction on the photothermal transport of PS nanoparticles with and without laser irradiation. (A) Schematic illustration of vitreous liquefaction over time (i.e., formation of liquefaction pockets (LP) and related collagen network (CN) degradation; (B) Time traces of average MSD for aged‐vitreous samples (0, 4, and 7 days post‐dissection) containing 520 nm‐ PS particles (dilution ratio 1/1000 (v/v)) without laser irradiation or ICG, based on individual particle traces summarized in Figure S7. (C) Evolution of the obtained fitting parameters, including diffusion coefficient *D* (left *y*‐axis) and confinement radius *r_c_* (right *y*‐axis), as a function of time after vitreous extraction. (D) Time traces of average MSD for laser‐irradiated (0.69 J/cm^2^, 532 nm) aged‐vitreous samples (0, 2, and 8 days post‐dissection) containing 520 nm‐ PS particles (dilution ratio 1/1000 (v/v)) and ICG (0.5 mg/mL), based on individual particle traces shown in Figure S8. E: Extracted fitting parameters: diffusion coefficient *D* (left *y*‐axis) and particle velocity *v* (right *y*‐axis) as a function of time after vitreous extraction under laser irradiation.

First, the motion of 520 nm PS nanoparticles was studied in vitreous samples aged 0, 4 (Video S11), and 7 days (Video S12) without ICG or laser irradiation. (Figure 6A) These videos revealed an increased random motion of PS nanoparticles in the absence of laser irradiation, likely due to a less confined environment resulting from progressive vitreous liquefaction over time. MPT analysis (Figure 6B) yielded diffusion constants that increase with time for samples aged 0, 4, and 7 days (0.029 ± 0.006, 0.038 ± 0.003, and 0.17 ± 0.005 µm^2^/s, respectively, Figure 6C). The determined confinement radius (see Methods) supports the presence of a degraded microenvironment in week‐old vitreous samples, exhibiting a radius of 4.5 ± 0.8 µm, approximately six times larger than that measured in freshly analyzed samples at day 0 (0.77 ± 0.06 µm). Previous studies have reported an average mesh size of 510–565 nm for native vitreous that remains intact within the eye [9, 10]. In our case, the observed higher radius values may result from mechanical deformation or structural relaxation of the vitreous matrix, related to liquefaction over time and extraction from the eye. Additionally, the slight negative surface charge of the particles may provide them with some extra mobility due to electrostatic repulsion with the vitreous matrix [11]

The motion of 520 nm PS nanoparticles was then assessed in aging vitreous (0, 2 (Video S13) and 8 days (Video S14)) in the presence of ICG (0.5 mg/mL) and upon laser irradiation (0.69 J/cm^2^, 532 nm) The results are summarized in Figure 6D, where the average MSD curves are plotted as a function of time. The MPT analysis revealed that, upon laser exposure, particles in 8‐day old vitreous samples (Video S14) follow a quadratic motion with a significant higher velocity (3.24 ± 0.76 µm/s, see Figure 6E) than the ones determined on day 0 or 2 (Video S13), being 1.25 ± 0.14 µm/s and 1.58 ± 0.05 µm/s, respectively (see Figure 6E), while the diffusion constant remains similar in all samples (3.97 ± 0.51, 2.9 ± 0.25, 4.34 ± 0.56 µm^2^/s for 8‐day, 2‐day, and 0‐day samples, respectively) as shown in Figure 6E.

These two experiments (i.e., without and with laser irradiation) demonstrate a significant effect of vitreous aging on the mobility of nanoparticles without laser irradiation (i.e., by an increase of the confinement radius) and with laser irradiation (i.e., by an increase of the particle's directional velocity), consistent with progressive vitreous liquefaction. It proves that vitreous liquefaction is a non‐negligible factor in the photothermal transport of nanoparticles in the vitreous. Importantly, however, liquefaction did not impede the guidance of particles under laser irradiation.

## 3 Discussion

In this study, we have demonstrated that the combined use of a light‐absorbing ocular dye (ICG) with nanosecond pulsed‐laser irradiation (2–5 ns) enables photothermal transport of PS nanoparticles in both water and bovine vitreous. The particles exhibit directed motion toward the laser focus, forming elongated trajectories, whereas in the absence of laser irradiation and/or ICG, their motion remains Brownian (random) and even more restricted in the tight matrix of the vitreous (confined motion). We observed particle attraction (for both the 520 nm and 1 µm PS particles) at the laser focus point in both media. Varying experimental parameters such as ICG concentration, laser fluence, particle diameter, and distance from the laser focus point also impacted the photothermal transport of the particles. The particle velocity can therefore be adjusted by changing the dye concentration. We identified that a minimal concentration of ICG (0.2 mg/mL) is needed to induce photothermal transport; at lower ICG concentrations, the PS nanoparticles only exhibited Brownian motion, while upon increasing the ICG concentration, transport of PS nanoparticles highlighted the crucial role of ICG. The laser fluence also plays a crucial role in the photothermal transport of nanoparticles, as a monotonic increase in particle velocity with fluence has been observed. While the mesh size of the collagen network in the vitreous is around 500 nm, the motion of larger particles (1 µm) seemed possible upon pulsed‐laser illumination in the presence of ICG. We also want to raise attention on the importance of the particle distance from the laser spot in photothermal transport. Beyond the successful transport of nanoparticles over long distances (up to nearly one millimeter) from the heating site, we observed enhanced particle diffusion near the laser focal point, resulting from localized heating and partial disruption of the vitreous network. We also demonstrated that the velocity tends to decrease as particles approach the laser focus, likely due to a high accumulation that restricts mobility in the progressively crowded environment. This observation suggests that excessive accumulation near the illuminated zone creates a local crowding that hinders further motion. Our analyses of the underlying parameters influencing photothermal transport of nanoparticles might form the basis for the fine‐tuning of NP motion in the vitreous in future medical applications. Furthermore, we observed that photothermal transport of nanoparticles in the vitreous is influenced by aging (liquefaction) of the vitreous, indicating that patient‐to‐patient variability (age, pathology, etc.) might impact the photothermal transport of particles.

Building on our empirical findings, we developed a numerical model that offers valuable insights into the mechanisms underlying photothermal transport. We found that the photothermal transport of nanoparticles in both water and the vitreous is governed by a combination of thermal convection and thermophoresis, with the relative contribution of each mechanism depending on the medium. We note that the sign of the Soret coefficient (S_T_) for PS nanoparticles is not universal. Although bare PS particles in simple aqueous solutions often exhibit S_T_ > 0 (motion toward colder regions), both the magnitude and sign of S_T_ are known to vary widely depending on the electrolyte composition, temperature, particle size, and surface chemistry [44, 45]. In our case, the use of ICG, an anionic and amphiphilic dye, likely alters the interfacial properties of PS particles, which could impact the sign of the S_T_. Furthermore, simulations performed with both positive and negative S_T_ (Figure 5C,D) revealed particle attraction toward the laser beam, indicating that the transport direction is independent of the sign of S_T_. This suggests that thermophoresis alone cannot account for the observed transport behavior in water and in the vitreous and that convection is likely the dominant mechanism. Indeed, in the vitreous, the thermophoretic velocity is markedly reduced due to its higher viscosity, while stronger convection flows emerge as a result of the greater sample thickness compared to water. Importantly, fluid velocity also strongly depends on the spatial position relative to the heat source and nearby boundaries. Although simulated velocities near the glass surface are lower in the vitreous than in water (Figure S6), experimental data reveal higher particle velocities in the vitreous (Figure 3G). This apparent discrepancy can be explained by the increased sample thickness in the vitreous (exceeding 500 µm), which supports more extensive convection flows and a longer‐range particle attraction toward the laser spot. For example, in Figure 3H, particles are drawn in from distances beyond 800 µm, suggesting a substantial vertical extent of the sample. Additionally, prolonged laser exposure (over 30 s) can lead to cumulative heating, resulting in significant local temperature increases and partial liquefaction of the vitreous, thereby reducing its viscosity and further enhancing fluid motion. While optothermal platforms, such as those demonstrated by Ding et al. [46] using PEG solutions with NIR irradiation often require a synthetic matrix to support particle manipulation, our method operates directly in the vitreous, a biologically relevant medium, without the need for additional synthetic substrate, therefore facilitating clinical translation. The use of a clinically‐approved ocular dye and laser modality further ensures biocompatibility and underlines its strong clinical potential. A controlled, localized transport is achieved through the interplay of pulsed laser heating and convection, opening the path for a precise and targeted delivery of particles in the eye. One limitation of our system compared to optothermal platforms is, however, that thermophoresis only contributes minimally to particle transport, making the process largely dependent on convection. As a result, precise control of particularly small particles or in heterogeneous regions of the vitreous can be more challenging compared to systems where thermophoretic forces play a larger role. Together, these results highlight the complex interplay of thermal convection, thermophoresis, and boundary conditions, all of which must be carefully considered when interpreting particle transport under opto‐thermal excitation, particularly for biomedical applications.

Our experimental and numerical findings prove to be promising for light‐mediated delivery in the posterior part of the eye, but further work is needed for future clinical implementation, especially regarding laser use and heat generation. In general, heat generation under laser irradiation is a cumulative process; over prolonged exposure, local temperatures progressively rise, leading to steeper thermal gradients and stronger fluid flows. In our simulations, the temperature increase shown in Figure 5A was calculated over a 10 ms period, corresponding to the 100 Hz laser repetition rate. When we extend the simulations to longer timescales, such as 0.1–1 s, the cumulative heating can elevate local temperatures to nearly 100°C, potentially leading to liquid boiling. However, in our experiments, boiling was rarely observed (only for very high laser fluences) because continuous fluid motion brings cooler liquid toward the laser spot, mitigating local temperature rise. Simulated heating can also be adjusted by varying the absorption coefficient (ICG concentration) or the laser power (fluence). This aligns with experimental results, where particle diffusion constants in water increase with higher ICG concentrations (Figure 2H), corresponding to local temperatures approaching 80°C at 0.5 mg/mL, or with increasing laser fluence (Figure 3G). In our study, a pulsed laser (2–5 ns; 532 nm) was used. A strong advantage of using this type of laser for ophthalmological purposes is that it is widely used in the clinic [47] and that our technology would not need extensive development of optics in case of clinical translation. However, safety concerns still need to be addressed. Although the bulk temperature remained nearly constant (i.e., around 19°C) during 120 s of laser irradiation (Figure S3), confirming negligible laser‐induced heating under our experimental parameters, potential phototoxicity may still arise from the dye itself. ICG has been approved for medical use by the Food and Drug Administration (FDA) since 1959. While our choice for this conceptual study relied on the clinical use of ICG, it is also known to induce radical oxygen species (ROS) upon light irradiation [48], which raises potential safety concerns, particularly to the retina. However, the lack of a consistent trend in experimental results on ICG toxicity [49, 50, 51] makes it challenging to draw any definitive general conclusion. Anyhow, to mitigate the toxicity risk of ICG, general safety guidelines have emerged, like reducing the dye concentration and the exposure time [52]. Reducing ICG concentration from 5 to 0.5 mg/mL^−1^ and the exposure time (less than 10 s) enabled surgeons to suppress visual field defects and retinal pigment epithelium (RPE) damage for inner limiting membrane (ILM) visualization [53]. We have demonstrated, in our approach, that optimal photothermal transport of nanoparticles can be reached for ICG concentrations starting at 0.2 mg/mL and using a pulse laser irradiation between 2 and 5 ns. In addition, another strategy to reduce potential ICG toxicity relies on increasing the osmolarity of the particle solution and/ or on modifying the dye composition. For example, it has been shown that using infracyanine green— a dye that is structurally close to ICG— dissolved in glucose 5 (w/v)% as an iso‐osmolar solution, significantly reduced toxicity to RPE cells [54]. Finally, laser irradiation can also be associated with potential toxicity risks related to the wavelength and exposure time, which are important to consider for potential clinical translation. Our study has been performed at 532 nm because this wavelength is commonly used with nanosecond lasers in the clinic (e.g., for iridotomy [47], laser trabeculoplasty (SLT) [55]). It is important to underline that 532 nm is not the maximum absorption peak of ICG (i.e., 780 nm [56]). Therefore, the laser irradiation of a mixture of ICG and PS nanoparticles at higher wavelengths could further stimulate the motion (i.e., diffusion and velocity) but could also lead to increased heat dissipation and potentially higher toxicity, since we have shown that laser heating increased linearly with the laser wavelength (Figure S9C). Important to note as well is that the use of 532 nm pulsed‐laser in the vitreous poses risks for the retina owing to its pigmented (hence light‐absorbing) nature. Some reports have indeed demonstrated that pulsed‐laser irradiation in the direct neighborhood of the retina could induce photomechanical damage [57]. Therefore, other dyes with limited ROS generation and absorption in the near infrared (NIR) II region could be considered to further improve the safety of our approach by limiting photomechanical damage. Finally, the laser fluence also plays an important role in safety. Based on the ANSI Z136.1 and ICNIRP standards, and following the approach of Delori et al. [58], we estimate the Maximum Permissible Exposure (MPE) for our laser parameters (λ = 532 nm, pulse duration = 2–5 ns, repetition rate = 100 Hz, beam diameter = 217.4 µm) to be on the order of 10^−^⁵ J/cm^2^ per pulse. As expected, our experimental fluences (0.69 and 1.03 J/cm^2^, see Table 1) are higher than this theoretical safety limit, which is defined for unintentional retinal exposure and includes large safety margins. Nevertheless, the fluence range used in this study remains within the same order of magnitude as those typically applied in clinical procedures using 532 nm Q‐switched Nd:YAG lasers (e.g., Ellex Tango Reflex), which operate at approximately 0.2–2 J/cm^2^ ^2^ [59, 60, 61]. We demonstrated that fluence values within this range (i.e., 0.69 and 1.03 J/cm^2^, see Table 1) are already sufficient to induce photothermal transport of PS nanoparticles in the presence of ICG in the vitreous. This suggests that effective photothermal transport can be achieved at clinically relevant fluences, potentially allowing for safe translation. However, further research is still needed to identify the optimal balance between safety and efficacy (i.e., reaching high velocity at minimal energy).

Given its significant physical and experimental insights, we believe this first study on the ‘directed’ photothermal transport of particles in the vitreous, using clinical laser settings and a clinically approved dye, might pave the way for light‐guided strategies to deliver intravitreally‐injected drugs to the posterior segment of the eye. Our approach offers interesting therapeutic perspectives to face the major challenges associated with the intravitreal injection of drugs and particles, e.g., an insufficient drug concentration in target tissues caused by random and non‐directed diffusion. By enabling more precise drug delivery, our strategy could ultimately reduce the need for frequent IVT injections, such as the bi‐monthly injections currently required for wet age‐related macular degeneration (AMD) treatment [62]. In doing so, it may not only improve patient compliance and therapeutic outcomes but also significantly lower healthcare costs by reducing the number of administrations of often expensive drugs. This study demonstrates for the first time that photothermal transport can be selectively and controllably generated within the vitreous using clinical‐grade dyes and laser parameters already established in ophthalmology. This selective induction of convection within a complex biological matrix, such as the vitreous, represents an important step toward spatially guided delivery of intravitreally injected drugs using light‐based methods.

## 4 Materials & Methods

### 4.1 Chemical Materials

Deionized water was obtained from a Milli‐Q water purification system (Millipore, Bedford, MA, USA). Indocyanine green (ICG) was purchased from U.S. Pharmacopeia (USP, United States). Fluorescent Negatively‐Charged Polystyrene (PS) Microspheres, Dragon Green (𝝀_ex_ = 480 nm, 𝝀_em_ = 520 nm) 0.50 and 1.0 µm were purchased from Bangs Laboratories, Inc.

### 4.2 Water Sample Preparation

Physical mixtures of PS nanoparticles (520 nm; 1 µm) and ICG were prepared by first diluting ICG in deionized water under vortexing (Vortex V‐1 plus, BioSan) for 10–20 s. Subsequently, PS nanoparticles were added to the ICG aqueous solutions (dilution ratios: 1/1000; 1/5000 (v/v)) and vortexed again for another 10–20 s. The volume of PS nanoparticles was adjusted according to the targeted dilution ratio, e.g., for 1/1000 (v/v), 1 µL of PS nanoparticles was added to 999 µL of the ICG aqueous solution. Two droplets (approximately 100 µL) of the PS nanoparticle‐ICG mixture were then placed on a microscope slide pre‐treated with an adhesive ring, then covered with a coverslip to minimize water evaporation during experiments. Nanosecond laser pulses (Ekspla N230‐100‐SCU‐2H OPO, 532 nm, 2–5 ns) were subsequently applied. See Video S15 (Table S1) as an example showing the full field of view.

### 4.3 Bovine Vitreous Extraction and Sample Preparation

Fresh bovine eyes were enucleated only a few hours after cows were slaughtered (slaughterhouse Vion Group, Zottegem, Belgium). The eyes were transported and kept in ice‐cold‐CO_2_‐independent medium until dissection. After removing all extra‐ocular connective tissue and disinfecting the eyes by soaking them in 20 % ethanol, the sclera was punctured with a 21G needle around 10 mm below the limbus. This hole next serves as an entry point for the scissors used to cut the eye into two parts. Since the vitreous has a very fragile structure, it was carefully removed from the anterior part using a flat brush and kept at 4°C before use. First, 300–500 µL of vitreous was carefully cut and placed on a 50 mm glass‐bottomed dish. Subsequently, 50 µL of the aqueous mixture of ICG and (520 nm or 1 µm) PS nanoparticles were injected into the vitreous sample using a 1 mL syringe equipped with a 21.5G needle. The sample was allowed to equilibrate for 5 min with a cover slip to minimize water evaporation during experiments prior to applying nanosecond laser pulses (Ekspla N230‐100‐SCU‐2H OPO, 532 nm, 2–5 ns). See Video S16 (Table S1) as an example with a full field of view and Figure S10 for a schematic representation of the sample preparation.

### 4.4 Bovine Vitreous Liquefaction and Sample Preparation

Fresh bovine eyes were enucleated within a few hours after slaughter (Vion Group slaughterhouse, Zottegem, Belgium). The eyes were transported and maintained in ice‐cold CO_2_‐independent medium until dissection. After removing all extra‐ocular connective tissues, the eyes were disinfected by immersion in 20 % ethanol. The sclera was then punctured with a 21G needle approximately 10 mm below the limbus, creating an entry point for scissors used to cut the eye into two parts. Because the vitreous has a very fragile structure, it was carefully detached from the anterior part using a flat brush and kept at 4°C until use. Aliquots of 300–500 µL of vitreous were collected at days 0, 2, 4, and 8, gently sectioned, and placed on 50 mm glass‐bottomed dishes.

### 4.5 Optical Setup and Camera Recording

An inverted microscope (TE2000‐E, Nikon ECLIPSE) equipped with a 10x objective (Nikon CFI Plan Fluor) was used to focus the nanosecond laser onto areas containing PS nanoparticles and ICG within the samples (Figure S11). Because PS nanoparticles scatter sufficient light, dark‐field microscopy was used for visualization. Samples were illuminated with nanosecond laser pulses at a wavelength of 532 nm, with a pulse duration of 2–5 ns (Ekspla N230‐100‐SCU‐2H OPO) and a repetition rate of 100 Hz. A beam expander (#GBE05‐A, Thorlabs) combined with an iris diaphragm (#D37SZ, Thorlabs) was used to adjust the diameter of the laser beam to 217.4 µm. The laser pulse energy was monitored by an energy meter (J‐25MB‐HE&LE, Energy Max‐USB/RS sensors, Coherent) synchronized with the pulsed laser and placed behind a beamsplitter (Figure S11). The laser fluence was determined from the measured energy and beam diameter using the following equation:

$$\begin{array}{ccc} & & Laser\;fluence\;\left(J.cm^{-2}\right)\\ & & \;=\left(Energy\;at\;the\;sample\;E_{sample}\;\left(J\right)\right)\\ & & \;/\left(\pi \times {\left(laser\;beam\;diameter\;\left(cm\right)/2\right)}^{2}\right) \end{array}$$

where E_sample_ = measured energy (J) × conversion factor.

The conversion factor accounts for optical losses along the path between the beamsplitter and the sample. The set‐up was configured to record videos before, during, and after laser illumination using a sCMOS camera (Photometrics Prime sCMOS, pixel size of 6.5 µm) controlled by Micro‐Manager software (Multi‐D acquisition mode). A 10x microscope objective was used, corresponding to an effective pixel size of 0.65 µm and a field of view of 1316 µm^2^. Videos of 30–60 s were recorded at a frame rate of 1 frame per second (fps) as shown in Videos S1–S16 with a playback speed of 10x.

### 4.6 Temperature Assessment During Laser Irradiation

To determine whether the laser exposure induced an increase in bulk temperature, temperature measurements were performed in bovine vitreous samples during laser irradiation (n = 3; three independent samples obtained after eye dissection and stored at 4°C prior to experimentation) in the presence of polystyrene (PS) particles (diameter = 520 nm, dilution 1:1000) and indocyanine green (ICG, 0.5 mg/mL). Laser irradiation was performed for up to 120 s at 532 nm with a fluence of 0.69 J/cm^2^ and a pulse duration of 2–5 ns. Vitreous samples were placed in a 96‐well microplate, and a temperature probe (Objective Heater Controller, BIOPTECH, USA) was inserted into each well to continuously record the temperature during laser exposure (120 s per well), as previously described by Teirlinck et al. [63]. The correct operation and calibration of the temperature probe were verified using ultrapure water up to 50°C.

### 4.7 Quantification of Particle Attraction After Laser Irradiation

Experiments were conducted in bovine vitreous samples containing polystyrene (PS) nanoparticles (diameter = 520 nm, 1/1000 dilution) and indocyanine green (ICG, 0.5 mg/mL). Samples were irradiated with the pulsed laser (532 nm, fluence 0.69 J/cm^2^, pulse duration 2–5 ns). A series of video frames was recorded before, during, and after laser irradiation in different regions of the sample (n = 3). Image analysis was performed using MATLAB to quantify mean gray values (average pixel intensity in grayscale images) within a 200 pixels × 200 pixels (130 µm × 130 µm) region centered around the laser spot.

### 4.8 Image Processing for Particle Tracking During Laser Displacement

Because the laser beam was co‐aligned with the imaging axis, the region of interest (ROI) translated within the camera's field of view as the laser spot was manually repositioned. As a result, the particle of interest did not remain in a fixed location in the raw videos (see Video S17). To generate a continuous visualization of the particle trajectory, the corresponding ROIs were cropped and reassembled into a single sequence. Briefly, individual frames containing the particle of interest were extracted and cropped around the ROI in ImageJ. The cropped frames were then concatenated using the *Concatenate* function (ImageJ, NIH) to produce a continuous time‐ordered sequence in which the particle remains centered, allowing visualization of its motion relative to the displaced laser beam. No additional filtering or image enhancement was applied.

### 4.9 Multi‐Particle Tracking (MPT) Analysis

Particle motion was analyzed using multi‐particle tracking algorithms [11, 42, 43, 44] applied to time‐lapse microscopy videos acquired under various experimental conditions. The 2D positions of PS nanoparticles were tracked frame‐by‐frame using Fiji extension^66^ based on ImageJ and a custom‐written MATLAB script, allowing for the reconstruction of individual particle trajectories over time. The mean square displacement for each trajectory was calculated using Equation (3). Depending on the observed motion type, different fitting models [47] were applied: Equation (4) was used for free Brownian motion (Figure 2E), Equation (5) for directed motion (Figure 2F), and Equation (6) for confined motion (Figure 3E). Ensemble‐averaged MSD curves were generated from multiple trajectories under each condition, and the resulting data were fitted accordingly. Based on the fitting, the key fitting parameters were extracted, including the diffusion coefficient *D*, confinement radius *r_c_*, and particle velocity *v*. Statistical error bars were derived from the fitting residuals across individual particle tracks. To estimate the local temperature from the measured diffusion coefficients, we used Equations (1) and (2) to generate a diffusion‐temperature calibration curve (Figure S9A), based on the temperature‐dependent dynamic viscosity of water^67^. Using this curve, the diffusion constants extracted from experimental data (Figure 2F) were mapped to their corresponding temperatures, as shown in Figure S9B.

### 4.10 Numerical Calculations of Photothermal Effects

Numerical simulations were performed using finite‐element solver COMSOL Multiphysics 5.6 according to our previously published methods [45, 46, 47, 48]. To investigate the photothermal effects induced by laser‐illuminated ICG in different media, including water and bovine vitreous, the simulation model coupled several different modules, including electromagnetic waves (EMW), heat transfer (HT), and computational fluid dynamics (CFD), to capture the relevant mechanisms driving nanoparticle motion. The laser was modeled as a Gaussian beam in paraxial approximation [49, 50] with an average power *P* = 100 mW, corresponding to a fluence of 2 J/cm^2^ over a 270 µm‐diameter spot (area ≈ 5.73×10^−8^ m^2^), yielding energy ∼1.15 mJ per pulse at repetition rate *f* = 100 Hz. This results in an average power of ∼115 mW, matching experimental conditions. To justify treating the pulsed laser as a continuous source, we computed the thermal impedance *Z = (ωC𝜅)^−1/2^* for the applied materials, where ω = 2π×100 Hz is the angular frequency of the laser pulses, *C* = 4.2 MJ/(m^3^·K) is the volumetric heat capacity of water, and 𝜅 = 0.6 W/(m·K) is its thermal conductivity. This gives *Z* ≈ 2.5×10*^−5^* K/W, leading to negligible per‐cycle temperature fluctuations (∼2.5 µK) relative to the total rise (∼45°C over 10 ms), thus validating the continuous‐wave approximation. The medium (refractive index *n* = 1.33) was modeled as a dielectric slab with a height *h* = 200 µm for water or 500 µm for vitreous, and a lateral width of 3 h. The dynamic viscosity of liquefied vitreous was assumed to be four times that of water, based on reported values: 3 mPa s for vitreous^68^ and 0.7 mPa s for water^67^ at 37°C. Laser absorption by ICG was simulated using an effective electrical conductivity σ = 0.5 S/m, chosen to match the experimental power (100 mW) and wavelength (532 nm) while ensuring that temperature rises remained below 100°C per pulse cycle (10 ms), as shown in Figure S8C. Absorption‐induced heating generated spatial temperature gradients, which were then used to calculate thermal convection using the Navier–Stokes equations for incompressible flow based on the applied boundary conditions and starting room temperature of 20°C.

In addition to thermal convection, we also calculate the contribution of thermophoresis independently, using the modeled temperature gradients ∇*T*. To compute thermophoretic velocity *v_tp_* of nanoparticles (Figure 5C,D), we use the following relation [21, 23, 27]:

(7) $$v_{tp}=-D_{T}\nabla T=-DS_{T}\nabla T$$

where *D_T_* is the thermophoretic diffusion coefficient, *D* is the Brownian diffusion coefficient, and *S_T_* = *D_T_* / *D* is the Soret coefficient. The Soret coefficient quantifies the strength and direction of particle movement in response to a temperature gradient and depends on many factors, including temperature, particle size, surface properties, solvent viscosity, and thermal capacitance [23, 27]. Thermophoretic particle motion was modeled based on these gradients using Equation (8), with the Soret coefficient: [32]

(8) $$S_{T}=\frac{D_{T}}{D}=-\frac{\varepsilon_{0}\varepsilon }{2\eta TD}\frac{2\kappa }{\kappa +\kappa_{p}}\left(1+\frac{\partial \ln\varepsilon }{\partial \ln T}\right)\zeta^{2}$$

where *ε_0_* is a vacuum permittivity, *ε* = 78 is a dielectric constant of the medium, *κ_p_* = 0.13 W/(m·K) is a thermal conductivity of the particle, ζ = −30 mV is a zeta potential of the particle and τ = ∂(ln ε)/∂(ln T) is a permittivity‐related term, which becomes 2 and −1.4 for the negative and positive Soret coefficients, respectively [35]. In our case, this equation results in negative *S_T_* = ‐3.5 1/K for water and −0.8 1/K for vitreous and positive *S_T_* = 0.5 1/K for water and 0.1 1/K for vitreous, reflecting stronger thermophoretic behavior in water due to lower viscosity. The simulations produced steady‐state temperature distributions, flow velocity fields, and thermophoretic velocity profiles (Figure 5), allowing for direct comparison of the dominant transport mechanisms in each medium.

## Author Contributions

F.S. conceived and designed the research; L.G., Y.U. and F.S. designed the experiments; L.G. and L.D.C. performed the experiments; Y.U. and L.D.C performed MPT analysis; Y.U. and F.B. developed the numerical model; L.G., Y.U. and F.S. analyzed the data and wrote the manuscript; F.S. supervised the project. All authors contributed to the discussion of the results and to the revision of the manuscript.

## Conflicts of Interest

The authors declare no conflicts of interest.

## Additional Information

Generative AI (ChatGPT4) has been used to improve English in the manuscript. After using this tool/service, the authors reviewed and edited the content as needed and took full responsibility for the content of the publication.

## Supporting information




**Supporting File**: advs73420‐sup‐0001‐SuppMat.docx.

**Supporting File**: advs73420‐sup‐0002‐SI Videos.zip.

## Data Availability

The data that support the findings of this study are available from the corresponding author upon reasonable request.

## References

[1] C. Y. Lee , Y. S. You , S. H. Lee , and H. Jung , “Tower Microneedle Minimizes Vitreal Reflux In Intravitreal Injection,” Biomedical Microdevices 15 (2013): 841–848, 10.1007/s10544-013-9771-y. 23666517

[2] M. J. Burton , J. Ramke , A. P. Marques , et al., “The Lancet Global Health Commission on Global Eye Health: Vision Beyond 2020,” The Lancet Global Health 9 (2021): e489–e551, 10.1016/S2214-109X(20)30488-5. 33607016 PMC7966694

[3] M. Wels , D. Roels , K. Raemdonck , S. C. De Smedt , and F. Sauvage , “Challenges And Strategies For The Delivery Of Biologics To The Cornea,” Journal of Controlled Release 333 (2021): 560–578, 10.1016/j.jconrel.2021.04.008. 33857565

[4] S. Fogli , M. Del Re , E. Rofi , C. Posarelli , M. Figus , and R. Danesi , “Clinical Pharmacology Of Intravitreal Anti‐VEGF Drugs,” Eye (London, England) 32 (2018): 1010–1020, 10.1038/s41433-018-0021-7. 29398697 PMC5997665

[5] R. Milston , M. C. Madigan , and J. Sebag , “Vitreous Floaters: Etiology, Diagnostics, And Management,” Survey of Ophthalmology 61 (2016): 211–227, 10.1016/j.survophthal.2015.11.008. 26679984

[6] J. Sebag , “Imaging Vitreous,” Eye Lond England 16 (2002): 429–439. 10.1038/sj.eye.6700201 12101450

[7] M. Levin and N. Cohen , “The Effects Of Aging On The Mechanical Properties Of The Vitreous,” Journal of Biomechanics 119 (2021): 110310, 10.1016/j.jbiomech.2021.110310. 33721627

[8] J. Sebag , “Ageing of the Vitreous,” Eye (London, England) 1 (1987): 254–262, 10.1038/eye.1987.45. 3308528

[9] F. Watts , L. E. Tan , C. G. Wilson , J. M. Girkin , M. Tassieri , and A. J. Wright , “Investigating The Micro‐Rheology Of The Vitreous Humor Using An Optically Trapped Local Probe,” Journal of Optics 16 (2013): 015301, 10.1088/2040-8978/16/1/015301.

[10] P. N. Bishop , “Structural Macromolecules And Supramolecular Organisation Of The Vitreous Gel,” Progress in Retinal and Eye Research 19 (2000): 323–344, 10.1016/S1350-9462(99)00016-6. 10749380

[11] Q. Xu , N. J. Boylan , J. S. Suk , et al., “Nanoparticle Diffusion In, And Microrheology Of, The Bovine Vitreous Ex Vivo,” Journal of Controlled Release 167 (2013): 76–84, 10.1016/j.jconrel.2013.01.018. 23369761 PMC3693951

[12] M. M. Le Goff and P. N. Bishop , “Adult Vitreous Structure And Postnatal Changes,” Eye Lond England 22 (2008): 1214–1222. 10.1038/eye.2008.21 18309340

[13] J. Park , P. M. Bungay , R. J. Lutz , et al., “Evaluation Of Coupled Convective–Diffusive Transport Of Drugs Administered By Intravitreal Injection And Controlled Release Implant,” Journal of Controlled Release 105 (2005): 279–295, 10.1016/j.jconrel.2005.03.010. 15896868

[14] K. Cholkar , A. Patel , A. D. Vadlapudi , and A. K. Mitra , “Novel Nanomicellar Formulation Approaches for Anterior and Posterior Segment Ocular Drug Delivery,” Nanomedicine 2 (2012): 82–95. 10.2174/1877912311202020082 PMC4232191 25400717

[15] N. Tasharrofi , M. Nourozi , and A. Marzban , “How Liposomes Pave The Way For Ocular Drug Delivery After Topical Administration,” Journal of Drug Delivery Science and Technology 67 (2022): 103045, 10.1016/j.jddst.2021.103045.

[16] W. Li , H. Vanluchene , L. Raes , et al., “Efficacy *Versus* Immunogenicity of LNP‐Mediated Delivery of mRNA and Self‐Amplifying RNA Upon Intravitreal Injection In The Mouse Eye,” Journal of Controlled Release 385 (2025): 114027, 10.1016/j.jconrel.2025.114027. 40659060

[17] B. Chen , M. Ding , H. Tan , et al., “Visible‐Light‐Driven TiO2@N‐Au Nanorobot Penetrating the Vitreous,” Applied Materials Today 27 (2022): 101455, 10.1016/j.apmt.2022.101455.

[18] Z. Wu , J. Troll , H.‐H. Jeong , et al., “A Swarm Of Slippery Micropropellers Penetrates The Vitreous Body Of The Eye,” Science Advances 4 (2018): aat4388, 10.1126/sciadv.aat4388. PMC6214640 30406201

[19] M. K , S. K , and S. D. George , “Colloidal Manipulation Through Plasmonic and Non‐plasmonic Laser‐Assisted Heating,” Laser & Photonics Reviews 17 (2023): 2300303, 10.1002/lpor.202300303.

[20] H. Ding , Z. Chen , C. Ponce , and Y. Zheng , “Optothermal Rotation Of Micro‐/Nano‐Objects,” Chemical Communications 59 (2023): 2208–2221, 10.1039/D2CC06955E. 36723196 PMC10189788

[21] L. Lin , E. H. Hill , X. Peng , and Y. Zheng , “Optothermal Manipulations of Colloidal Particles and Living Cells,” Accounts of Chemical Research 51 (2018): 1465–1474, 10.1021/acs.accounts.8b00102. 29799720 PMC6008228

[22] C. M. Jin , W. Lee , D. Kim , T. Kang , and I. Choi , “Photothermal Convection Lithography for Rapid and Direct Assembly of Colloidal Plasmonic Nanoparticles on Generic Substrates,” Small 14 (2018): 1803055, 10.1002/smll.201803055. 30294867

[23] S. Liu , L. Lin , and H.‐B. Sun , “Opto‐Thermophoretic Manipulation,” ACS Nano 15 (2021): 5925–5943, 10.1021/acsnano.0c10427. 33734695

[24] Y. Xie , C. Zhao , Y. Zhao , et al., “Optoacoustic Tweezers: A Programmable, Localized Cell Concentrator Based On Opto‐Thermally Generated, Acoustically Activated, Surface Bubbles,” Lab on a Chip 13 (2013): 1772–1779, 10.1039/c3lc00043e. 23511348 PMC3988908

[25] L. Lin , X. Peng , Z. Mao , X. Wei , C. Xie , and Y. Zheng , “Interfacial‐Entropy‐Driven Thermophoretic Tweezers,” Lab on a Chip 17 (2017): 3061–3070, 10.1039/C7LC00432J. 28805878

[26] D. Braun and A. Libchaber , “Trapping of DNA by Thermophoretic Depletion And Convection,” Physical Review Letters 89 (2002): 188103, 10.1103/PhysRevLett.89.188103. 12398641

[27] Y. Kim , H. Ding , and Y. Zheng , “Investigating Water/Oil Interfaces With Opto‐Thermophoresis,” Nature Communications 13 (2022): 3742, 10.1038/s41467-022-31546-3. PMC9243056 35768421

[28] N. Kavokine , S. Zou , R. Liu , A. Niguès , B. Zou , and L. Bocquet , “Ultrafast Photomechanical Transduction Through Thermophoretic Implosion,” Nature Communications 11 (2020): 50, 10.1038/s41467-019-13912-w. PMC6940389 31898691

[29] E. H. Hill , J. Li , L. Lin , Y. Liu , and Y. Zheng , “Opto‐Thermophoretic Attraction, Trapping, and Dynamic Manipulation of Lipid Vesicles,” Langmuir 34 (2018): 13252–13262, 10.1021/acs.langmuir.8b01979. 30350700 PMC6246038

[30] F. Aguilera Teba , A. Mohr , C. Eckardt , et al., “Trypan Blue Staining In Vitreoretinal Surgery,” Ophthalmology 110 (2003): 2409–2412, 10.1016/S0161-6420(03)00716-4. 14644726

[31] E. B. Rodrigues , C. H. Meyer , M. E. Farah , and P. Kroll , “Intravitreal Staining Of The Internal Limiting Membrane Using Indocyanine Green In The Treatment Of Macular Holes,” Ophthalmologica 219 (2005): 251–262. 16123549 10.1159/000086107

[32] T. Desmettre , J. M. Devoisselle , and S. Mordon , “Fluorescence Properties and Metabolic Features of Indocyanine Green (ICG) as Related to Angiography,” Survey of Ophthalmology 45 (2000): 15–27, 10.1016/S0039-6257(00)00123-5. 10946079

[33] R. W. Flower , “Evolution Of Indocyanine Green Dye Choroidal Angiography,” Optical Engineering 34 (1995): 727–736, 10.1117/12.191810.

[34] F. Sauvage , V. P. Nguyen , Y. Li , et al., “Laser‐Induced Nanobubbles Safely Ablate Vitreous Opacities In Vivo,” Nature Nanotechnology 17 (2022): 552–559, 10.1038/s41565-022-01086-4. 35302088

[35] K. de Bruin , N. Ruthardt , K. von Gersdorff , et al., “Cellular Dynamics of EGF Receptor–Targeted Synthetic Viruses,” Molecular Therapy 15 (2007): 1297–1305, 10.1038/sj.mt.6300176. 17457321

[36] Í. Amer Cid , Y. Y. Ussembayev , K. Neyts , and F. Strubbe , “Measurement Of The Amplitude And Phase Of The Electrophoretic And Electroosmotic Mobility Based On Fast Single‐Particle Tracking,” Electrophoresis 42 (2021): 1623–1635, 10.1002/elps.202100030. 34028056 PMC8454018

[37] C. Kettmayer , E. Gratton , and L. C. Estrada , “Comparison of MSD Analysis From Single particle Tracking With MSD From images. Getting the best of both worlds,” Methods and Applications in Fluorescence 12 (2023): 015001. 10.1088/2050-6120/acfd7e 37751748

[38] Y. Y. Ussembayev , N. K. Zawacka , F. Strubbe , Z. Hens , and K. Neyts , “Waveguiding of Photoluminescence in a Layer of Semiconductor Nanoparticles,” Nanomaterials 11 (2021): 683, 10.3390/nano11030683. 33803391 PMC7999844

[39] C. Rodà , B. B. V. Salzmann , I. Wagner , et al., “Stimulated Emission Through an Electron–Hole Plasma in Colloidal CdSe Quantum Rings,” Nano Letters 21 (2021): 10062–10069, 10.1021/acs.nanolett.1c03501. 34842440 PMC9113625

[40] Y. Ussembayev , F. Beunis , L. Oorlynck , M. Bahrami , F. Strubbe , and K. Neyts , “Single Elementary Charge Fluctuations on Nanoparticles in Aqueous Solution,” ACS Nano 17 (2023): 22952–22959, 10.1021/acsnano.3c08161. 37787115

[41] Y. Y. Ussembayev , Z. Hens , and K. Neyts , “Contrasting Anisotropy of Light Absorption and Emission by Semiconductor Nanoparticles,” ACS Photonics 6 (2019): 1146–1152, 10.1021/acsphotonics.8b01405.

[42] X. Peng , L. Lin , E. H. Hill , P. Kunal , S. M. Humphrey , and Y. Zheng , “Optothermophoretic Manipulation of Colloidal Particles in Nonionic Liquids,” The Journal of Physical Chemistry C 122 (2018): 24226–24234, 10.1021/acs.jpcc.8b03828. PMC6369910 30766650

[43] L. Lin , X. Peng , X. Wei , Z. Mao , C. Xie , and Y. Zheng , “Thermophoretic Tweezers for Low‐Power and Versatile Manipulation of Biological Cells,” ACS Nano 11 (2017): 3147–3154, 10.1021/acsnano.7b00207. 28230355

[44] A. Würger , “Transport in Charged Colloids Driven by Thermoelectricity,” Physical Review Letter 101 (2008): 108302. 10.1103/PhysRevLett.101.108302 18851262

[45] O. Syshchyk , D. Afanasenkau , Z. Wang , H. Kriegs , J. Buitenhuis , and S. Wiegand , “Influence Of Temperature And Charge Effects On Thermophoresis Of Polystyrene Beads⋆,” The European Physical Journal E 39 (2016): 129, 10.1140/epje/i2016-16129-y. 28000048

[46] H. Ding , Z. Chen , P. S. Kollipara , et al., “Programmable Multimodal Optothermal Manipulation of Synthetic Particles and Biological Cells,” ACS Nano 16 (2022): 10878–10889, 10.1021/acsnano.2c03111. 35816157 PMC9901196

[47] A. F. Borkenstein and E.‐M. Borkenstein , “Neodymium‐Doped Yttrium Aluminum Garnet (Nd: YAG) Laser Treatment in Ophthalmology: A Review of the Most Common Procedures Capsulotomy and Iridotomy,” Lasers in Medical Science 39 (2024): 167, 10.1007/s10103-024-04118-8. 38954050

[48] D. Atta , A. Elarif , and M. Al Bahrawy , “Reactive Oxygen Species Creation By Laser‐Irradiated Indocyanine Green As Photodynamic Therapy Modality: An In Vitro Study,” Lasers in Medical Science 38 (2023): 213, 10.1007/s10103-023-03876-1. 37704871 PMC10499713

[49] A. Gandorfer , C. Haritoglou , and A. Kampik , “Toxicity Of Indocyanine Green In Vitreoretinal Surgery,” Developments in Ophthalmology 42 (2008): 69–81. 18535381 10.1159/000138974

[50] J. S. Gale , A. A. Proulx , J. R. Gonder , A. J. Mao , and C. M. L. Hutnik , “Comparison Of The In Vitro Toxicity Of Indocyanine Green To That Of Trypan Blue In Human Retinal Pigment Epithelium Cell Cultures,” American Journal of Ophthalmology 138 (2004): 64–69, 10.1016/j.ajo.2004.02.061. 15234283

[51] S. Balaiya , V. S. Brar , R. K. Murthy , and K. Chalam , “Effects Of Indocyanine Green On Cultured Retinal Ganglion Cells In‐Vitro,” BMC Research Notes 2 (2009): 236, 10.1186/1756-0500-2-236. 19939252 PMC2791769

[52] D. Stanescu‐Segall and T. L. Jackson , “Vital Staining With Indocyanine Green: A Review Of The Clinical And Experimental Studies Relating To Safety,” Eye (London, England) 23 (2009): 504–518, 10.1038/eye.2008.249. 18670454

[53] F. Ando , K. Sasano , F. Suzuki , and N. Ohba , “Indocyanine Green‐Assisted ILM Peeling In Macular Hole Surgery Revisited,” American Journal of Ophthalmology 138 (2004): 886–887, 10.1016/j.ajo.2004.06.029. 15531340

[54] P. Stalmans , E. H. Van Aken , M. Veckeneer , E. J. Feron , and I. Stalmans , “Toxic Effect Of Indocyanine Green On Retinal Pigment Epithelium Related To Osmotic Effects Of The Solvent,” American Journal of Ophthalmology 134 (2002): 282–285, 10.1016/S0002-9394(02)01468-X. 12140045

[55] M. A. Latina , S. A. Sibayan , D. H. Shin , R. J. Noecker , and G. Marcellino , “Q‐Switched 532‐nm Nd:YAG Laser Trabeculoplasty (Selective Laser Trabeculoplasty),” Ophthalmology 105 (1998): 2082–2090, 10.1016/S0161-6420(98)91129-0. 9818610

[56] B. Yuan , N. Chen , and Q. Zhu , “Emission And Absorption Properties Of Indocyanine Green In Intralipid Solution,” Journal of Biomedical Optics 9 (2004): 497–503, 10.1117/1.1695411. 15189087 PMC1533769

[57] R. Brinkmann , G. Hüttmann , J. Rögener , J. Roider , R. Birngruber , and C. P. Lin , “Origin Of Retinal Pigment Epithelium Cell Damage By Pulsed Laser Irradiance In The Nanosecond To Microsecond Time Regimen,” Lasers in Surgery and Medicine 27 (2000): 451–464, 10.1002/1096-9101(2000)27:5<451::AID-LSM1006>3.0.CO;2-1. 11126439

[58] F. C. Delori , R. H. Webb , and D. H. Sliney , “Maximum Permissible Exposures for Ocular Safety (ANSI 2000), With Emphasis On Ophthalmic Devices,” Journal of the Optical Society of America A 24 (2007): 1250–1265, 10.1364/JOSAA.24.001250. 17429471

[59] Tango Reflex Multi‐Modality YAG/SLT Laser, https://www.laservision.nl/wp‐content/uploads/2024/03/Brochure‐Ellex‐Tango‐Reflex‐Neo.pdf.

[60] Z. S. Sacks , M. Dobkin‐Bekman , N. Geffen , M. Goldenfeld , and M. Belkin , “Non‐Contact Direct Selective Laser Trabeculoplasty: Light Propagation Analysis,” Biomedical Optics Express 11 (2020): 2889–2904, 10.1364/BOE.390849. 32637231 PMC7316017

[61] K. E. Leahy and A. J. White , “Selective Laser Trabeculoplasty: Current Perspectives,” Clinical Ophthalmology 9 (2015): 833–841. 26005327 10.2147/OPTH.S53490 PMC4433047

[62] M. Dang and M. S. Shoichet , “Long‐Acting Ocular Injectables: Are We Looking In The Right Direction?,” Advanced Science 11 (2024): 2306463. 38018313 10.1002/advs.202306463 PMC10885661

[63] E. Teirlinck , R. Xiong , T. Brans , et al., “Laser‐Induced Vapour Nanobubbles Improve Drug Diffusion And Efficiency In Bacterial Biofilms,” Nature Communications 9 (2018): 4518, 10.1038/s41467-018-06884-w. PMC6207769 30375378

